# Assessment of the transcriptional and cellular effects of CRISPRa targeting Dopa decarboxylase and GTP cyclohydrolase 1 in HEK293 and SH-SY5Y cellular models of Parkinsonian neurotoxicity

**DOI:** 10.3389/fncel.2026.1883789

**Published:** 2026-10-08

**Authors:** Siyethaba Zinhle Bhengu, Ravesh Singh, Veron Ramsuran, Zama Ndlondlo Princess Msibi

**Affiliations:** 1 School of Medicine, University of KwaZulu-Natal, Durban, South Africa; 2 National Health Laboratory Services, Inkosi Albert Luthuli Central Hospital, Durban, South Africa; 3 School of Medicine, University of KwaZulu-Natal, Howard College, Durban, South Africa

**Keywords:** 6-OHDA, CRISPRa, DDC, dopamine, GCH1, Parkinson's disease

## Abstract

Parkinson's disease (PD) is the second most common neurodegenerative disorder and is characterized by the progressive loss of dopaminergic neurons in the substantia nigra pars compacta (SNc), leading to dopamine deficiency and motor dysfunction. Clustered regularly interspaced palindromic repeats (CRISPR)-based technologies provide a potential approach for targeted gene modulation, although their effectiveness and safety in cellular models of PD remain to be established. This study investigated the feasibility and transcriptional effects of CRISPR activation (CRISPRa) targeting Dopa decarboxylase (*DDC*) and GTP cyclohydrolase 1 (*GCH1*) in human embryonic kidney (HEK293) cells and a 6-hydroxydopamine (6-OHDA)-induced neurotoxicity model using human neuroblastoma (SH-SY5Y) cells. Parkinsonian-like toxicity was induced in SH-SY5Y cells with 6-OHDA prior to CRISPRa transfection. A vector-free CRISPRa system was used to target *DDC* and *GCH1*, with single-guide RNAs (sgRNAs) assembled with dCas9-VP64 and MS2-p65-HSF1 (MPH) to form ribonucleoprotein (RNP) complexes and delivered using Lipofectamine 3000. CRISPRa targeting of *DDC* was associated with increased *DDC* expression in SH-SY5Y cells relative to untreated controls at 24 h (*p* = 0.0496) and 48 h (*p* = 0.0064). However, expression levels did not differ significantly from those observed in the 6-OHDA-treated PD control group. In contrast, *GCH1* expression was not significantly altered following CRISPRa. CRISPRa also had no significant effect on cell viability in HEK293 cells. In SH-SY5Y cells, 6-OHDA significantly reduced cell viability compared with untreated controls (*p* = 0.0129). Similar reductions were observed in *DDC*-targeted cells at 24 h (*p* = 0.0026) and 48 h (*p* = 0.0017), and in *GCH1*-targeted cells at 24 h (*p* = 0.0017) and 48 h (*p* = 0.0008); however, none of the CRISPRa-treated groups differed significantly from the 6-OHDA-treated PD control. These findings indicate that CRISPRa elicited a modest transcriptional response, characterized by upregulation of *DDC* without detectable modulation of *GCH1*, and did not exacerbate 6-OHDA-induced cytotoxicity. Further optimisation, together with protein-level, enzymatic activity, and dopamine measurements, is required to determine whether these transcriptional changes translate into functional effects on dopamine biosynthesis.

## 1 Introduction

Parkinson's disease (PD) is the second most common neurodegenerative disorder worldwide and affects an estimated 11.7 million people globally. The prevalence of PD is expected to increase further owing to population aging, making it a growing public health concern (Deliz et al., 2024; Singh et al., 2022; Tysnes and Storstein, 2017). PD is characterized by the progressive loss of dopaminergic neurons in the substantia nigra pars compacta (SNc), resulting in dopamine deficiency and the development of motor symptoms such as bradykinesia, rigidity, and resting tremor. Although the pathological mechanisms of PD are complex and involve processes such as α-synuclein (α-syn) aggregation, mitochondrial dysfunction, and oxidative stress, dopamine depletion remains central to the manifestation of motor dysfunction and therefore a primary therapeutic target (Espay and McFarthing, 2023; Grigoletto and Colla, 2022; Cabestany and Bayés, 2023).

Levodopa (L-dopa), the metabolic precursor of dopamine, remains the gold-standard treatment for PD motor symptoms. Following administration, L-dopa is converted to dopamine by aromatic L-amino acid decarboxylase (AADC), encoded by the *Dopa decarboxylase* (*DDC*) gene. Although L-dopa provides significant symptomatic benefit, its long-term use is associated with motor complications, and its effectiveness ultimately depends on the functionality of surviving dopaminergic neurons (Figge et al., 2024; Del Bello et al., 2019; Lane, 2019). These limitations have motivated the development of strategies aimed at enhancing endogenous dopamine biosynthesis and dopaminergic function.

Dopamine synthesis depends on the coordinated activity of several enzymes and cofactors. *DDC* encodes AADC, which catalyzes the conversion of L-dopa to dopamine, whereas *GTP cyclohydrolase 1* (*GCH1*) encodes the rate-limiting enzyme in tetrahydrobiopterin (BH_4_) biosynthesis. As BH_4_ is an essential cofactor for tyrosine hydroxylase (TH), *GCH1* indirectly supports dopamine production (Sportelli et al., 2024; Zahoor et al., 2018). Dysregulation of dopamine biosynthetic pathways has been associated with impaired dopaminergic function and altered responses to L-dopa therapy in PD (Tuominen and Renko, 2024). Given their complementary roles in dopamine biosynthesis, *DDC* and *GCH1* represent biologically relevant targets for therapeutic modulation aimed at enhancing endogenous dopamine production, making them attractive candidates for clustered regularly interspaced palindromic repeats activation (CRISPRa)-mediated gene activation.

Gene therapy approaches targeting dopamine-related pathways have shown promising results in preclinical and clinical studies (Susanna et al., 2022; Pearson et al., 2021; Muramatsu et al., 2002). However, conventional gene-replacement strategies require the delivery and sustained expression of exogenous genetic material and may be limited by issues including vector capacity, immunogenicity, manufacturing costs, and control of transgene expression (Okada and Ortiz, 2022; Baskin et al., 2021; Martier and Konstantinova, 2020). CRISPRa has emerged as an alternative strategy that employs catalytically inactive Cas9 (dCas9) fused to transcriptional activators to increase expression of endogenous genes without introducing deoxyribonucleic acid (DNA) double-strand breaks (López-Beltrán et al., 2024; Bhatia and Pooja, 2023). By exploiting native genomic regulatory elements, CRISPRa offers a potentially flexible approach for targeted transcriptional enhancement.

Despite these advantages, the application of CRISPRa to dopamine biosynthetic genes relevant to PD remains poorly characterized. In particular, it is unclear whether CRISPRa can effectively modulate endogenous *DDC* and *GCH1* expression in cellular models relevant to dopaminergic dysfunction and neurotoxic stress, or whether such modulation affects cellular viability. To address this knowledge gap, the present study investigated CRISPRa-mediated activation of *DDC* and *GCH1* in HEK293 cells and in 6-hydroxydopamine (6-OHDA)-treated SH-SY5Y cells, a widely used model of Parkinsonian neurotoxicity (Kovalevich and Langford, 2013). HEK293 cells were included as a non-neuronal comparison model because of its robust transfection and growth efficiency, while SH-SY5Y cells were used to assess CRISPRa responses under PD-relevant neurotoxic conditions. In addition to evaluating gene expression, cellular viability was assessed to determine whether CRISPRa-mediated targeting of dopamine biosynthetic genes was associated with adverse cellular effects. This study therefore provides an initial assessment of the feasibility and cellular consequences of CRISPRa-mediated regulation of *DDC* and *GCH1* in models relevant to PD.

## 2 Materials and methods

### 2.1 Ethical considerations

Ethical clearance for the study was obtained from the Biomedical Research Ethics Committee (BREC) of the University of KwaZulu-Natal (Reference number BREC/00006274/2023).

### 2.2 Cell culture

SH-SY5Y and HEK293 cell lines were obtained from the Department of Biochemistry, University of KwaZulu-Natal, South Africa. SH-SY5Y cells are derived from a human neuroblastoma cell line and exhibit neuronal-like characteristics, whereas HEK293 cells are derived from human embryonic kidney cells and provide a non-neuronal cell model commonly used in molecular and transfection studies. In the present study, SH-SY5Y cells were used as a neuronal-like model for evaluating CRISPRa-mediated transcriptional responses under 6-hydroxydopamine bromide (6-OHDA)-induced Parkinsonian-like conditions, whereas HEK293 cells were included as a non-neuronal comparison model to assess cellular viability following CRISPRa treatment. Cells were cultured under standard conditions at 37 °C in a humidified incubator containing 5% CO_2_. Frozen cell stocks were rapidly thawed at 37 °C and centrifuged at 400 rpm for 10 min at room temperature. The resulting cell pellets were resuspended in 4 mL of pre-warmed complete growth medium to remove residual dimethyl sulfoxide (DMSO). SH-SY5Y cells were maintained in Dulbecco's modified Eagle's medium/Ham-12 (DMEM/F-12) supplemented with 10% Fetal bovine serum (FBS) and 1% penicillin–streptomycin (Sigma-Aldrich, South Africa), whereas HEK293 cells were cultured in Dulbecco's modified Eagle's medium (DMEM) supplemented with 10% FBS, 1% L-glutamine, and 1% penicillin–streptomycin (Sigma-Aldrich, South Africa). Cells were seeded into T25 culture flasks and allowed to grow to 80–90% confluency. For passaging, the culture medium was removed, followed by a three-step wash with sterile phosphate buffered saline (PBS). Subsequently, 2 mL of Trypsin–EDTA was added, and cells were incubated at 37 °C for 2 min. Trypsin activity was neutralized with complete medium, and cells were transferred to T75 flasks containing 10 mL of fresh medium and gently agitated to ensure uniform distribution. SH-SY5Y cells were passaged twice weekly, with medium changes every 48 h, whereas HEK293 cells were passaged as required, with culture medium replaced every 2–3 days.

### 2.3 Experimental design

Both SH-SY5Y and HEK293 cells were cultured under standard conditions at 37 °C in a humidified atmosphere containing 5% CO_2_ until 80–90% confluency was reached. Cells were subsequently seeded into six-well plates at a density of 2 × 10^5^ cells per well according to the respective experimental groups. For HEK293 cells, the experimental groups consisted of: (i) untreated control (Control); (ii) CRISPRa targeting *DDC*, assessed 24 h after transfection (CRISPRa+*DDC*, 24 h); (iii) CRISPRa targeting *DDC*, assessed 48 h after transfection (CRISPRa+*DDC*, 48 h); (iv) CRISPRa targeting *GCH1*, assessed 24 h after transfection (CRISPRa+*GCH1*, 24 h); and (v) CRISPRa targeting *GCH1*, assessed 48 h after transfection (CRISPRa+*GCH1*, 48 h). For SH-SY5Y cells, the experimental groups consisted of: (i) untreated control (Control); (ii) cells treated with 6-OHDA (PD control); (iii) 6-OHDA-treated cells transfected with CRISPRa targeting *DDC* and assessed 24 h after transfection (6-OHDA+CRISPRa+ *DDC*, 24 h); (iv) 6-OHDA-treated cells transfected with CRISPRa targeting *DDC* and assessed 48 h after transfection (6-OHDA+CRISPRa+ *DDC*, 48 h); (v) 6-OHDA-treated cells transfected with CRISPRa targeting *GCH1* and assessed 24 h after transfection (6-OHDA+CRISPRa+*GCH1*, 24 h); and (vi) 6-OHDA-treated cells transfected with CRISPRa targeting *GCH1* and assessed 48 h after transfection (6-OHDA+CRISPRa+*GCH1*, 48 h). For the SH-SY5Y Parkinsonian-like model, cells were treated with 6-OHDA and incubated for 24 h before CRISPRa transfection. At the time of transfection, both HEK293 and SH-SY5Y cells had reached approximately 70% confluency. Following transfection, cells were maintained for either 24 or 48 h according to the experimental group. Three independent biological replicates (*n* = 3) were included per group. Cells were subsequently assessed for viability and target gene expression.

### 2.4 PD model induction

SH-SY5Y cells were used to establish an *in vitro* model of PD. Parkinsonian-like toxicity was induced using 6-OHDA (Sigma-Aldrich, South Africa). Briefly, a fresh 6-OHDA solution was prepared by dissolving 0.2% ascorbic acid in 0.9% saline, after which 6-OHDA was added to obtain a final concentration of 0.04 μg/μL. The solution was kept on ice and protected from light until use. Cells were treated with 25 μM 6-OHDA for 1 h at 37 °C. After treatment, the solution containing 6-OHDA was removed and replaced with fresh complete medium. The cells were then incubated for an additional 24 h at 37 °C before further experiments.

### 2.5 CRISPRa design and transfection

#### 2.5.1 sgRNA design and in vitro transcription

sgRNAs targeting *DDC* and *GCH1* were designed using the OmniGuide RNA design tool (GenScript, USA), with guide sequences specifically selected to target promoter and regulatory regions to promote transcriptional activation. A single sgRNA was selected for *DDC* (5′-ACGTGGAGCCCGGGTACCTG-3′), while a dual sgRNA strategy was employed for *GCH1*, targeting both promoter (5′-TCGTTATCCTCCTCGCTGCG-3′) and enhancer (5′-GCTCGTTATCCTCCTCGCTG-3′) regions to enhance activation efficiency. For sgRNA synthesis, complementary forward and reverse oligonucleotides containing a T7 promoter sequence were resuspended to 100 μM, combined in equimolar ratios, and annealed by heating to 95 °C for 5 min followed by gradual cooling to 25 °C to generate dsDNA templates. These templates were subsequently used for *in vitro* transcription in 20 μL reactions containing T7 RNA polymerase, transcription buffer, and nucleotide triphosphates, with incubation at 37 °C for 4 h. Following transcription, residual DNA templates were removed by DNase I treatment at 37 °C for 15 min. The resulting sgRNAs were purified using column-based RNA purification, eluted in RNase-free water, and quantified spectrophotometrically. sgRNA concentration and purity were assessed using a NanoDrop™ 2000 spectrophotometer (Thermo Scientific, Roche, South Africa), yielding an A260/A280 ratio of approximately 2.0, indicative of high RNA purity suitable for downstream applications, after which sgRNAs were aliquoted and stored at −80 °C until further use. The sgRNAs were subsequently used with a dCas9-VP64/MS2-p65-HSF1 (MPH) CRISPRa system to promote transcriptional activation of the target genes.

#### 2.5.2 CRISPRa RNP complex assembly

CRISPRa complexes were assembled separately for each target gene using a stepwise procedure. For *DDC* activation, dCas9-VP64 protein (15 pmol) was first combined with *DDC*-specific sgRNA (30 pmol) and incubated at room temperature for 10 min to facilitate formation of the dCas9-VP64–sgRNA complex. MS2-p65-HSF1 (MPH) activator protein (15 pmol) was subsequently added, followed by a further 10-min incubation at room temperature to complete CRISPRa complex assembly. The final assembled *DDC* CRISPRa RNP complex volume was 1.65 μL per well, and this volume was used for subsequent Lipofectamine complexation.

For *GCH1* activation, a dual-sgRNA strategy was used. dCas9-VP64 protein (15 pmol) was first combined with *GCH1* sgRNA #1 (30 pmol) and incubated at room temperature for 10 min. *GCH1* sgRNA #2 (30 pmol) was then added, followed by a further 5-min incubation at room temperature. MS2-p65-HSF1 (MPH) activator protein (15 pmol) was subsequently added and incubated for an additional 10 min at room temperature to complete CRISPRa complex assembly. The final assembled *GCH1* CRISPRa RNP complex volume was 1.95 μL per well, and this volume was used for subsequent Lipofectamine complexation. Following assembly, the respective CRISPRa complexes were maintained on ice until Lipofectamine complexation and subsequent transfection.

#### 2.5.3 Lipofectamine complex preparation and transfection

Cells were transfected once they reached approximately 70% confluency. For transfection, *DDC* and *GCH1* CRISPRa complexes were prepared separately. For the *DDC* condition, 100 μL Opti-MEM was combined with 6.6 μL of the *DDC* RNP complex (1.65 μL per well) and 10 μL P3000 Enhancer (2.5 μL per well) for four wells. For the *GCH1* condition, 100 μL Opti-MEM was combined with 7.8 μL of the *GCH1* RNP complex (1.95 μL per well) and 10 μL P3000 Enhancer (2.5 μL per well). Each mixture was gently mixed and incubated at room temperature for 5 min. Separately, 15 μL Lipofectamine 3000 (3.75 μL per well) was diluted in 100 μL Opti-MEM for each condition and incubated at room temperature for 5 min. The diluted Lipofectamine 3000 was then added to the corresponding *DDC* or *GCH1* RNP mixture, gently mixed by pipetting 3–5 times, and incubated at room temperature for 15–20 min to allow complex formation. Subsequently, 50 μL of the respective transfection complex was added to each well. Control wells received 50 μL Opti-MEM without RNP. The plates were gently rocked to distribute the complexes evenly and incubated at 37 °C with 5% CO_2_ for 4 h. Following transfection, the transfection medium was removed, and the cells were gently washed with PBS where appropriate. Cells were then maintained in 500 μL complete culture medium consisting of DMEM/F-12 supplemented with 10% FBS, 1% non-essential amino acids (NEAA), 1% penicillin-streptomycin, and 1 × B-27 Supplement, and returned to the incubator for the designated experimental period.

### 2.6 Cell viability assay

Cell viability was assessed using the MTT assay. SH-SY5Y and HEK293 cells were incubated with 120 μL of MTT-containing medium for 3 h at 37 °C. Medium was then removed, 100 μL of dimethyl sulfoxide (DMSO) was added to dissolve the crystals, and plates were incubated for 1 h at 37 °C. Absorbance was measured at 590 nm using a microplate reader (UV Vis Spectrophotometer, USA).

### 2.7 Gene expression

Total RNA was extracted using TRIzol™ reagent (Thermo Fisher Scientific, South Africa) according to the manufacturer's phenol–chloroform extraction protocol. Briefly, culture medium was removed from wells containing the respective experimental groups for both cell lines, and cells were washed with PBS to remove residual medium. Cells were lysed directly in 500 μL TRIzol™ reagent per well and homogenized by vigorous pipetting to facilitate cellular disruption and dissociation of nucleoprotein complexes. Lysates were incubated at room temperature for 5 min, followed by the addition of chloroform to induce phase separation. Samples were vortexed and centrifuged at 12,000 × *g* for 15 min at 4 °C. The upper RNA-containing aqueous phase was carefully transferred to a fresh RNase-free tube. RNA was precipitated by adding isopropanol and incubating at room temperature for 10 min, followed by centrifugation at 12,000 × *g* for 10 min at 4 °C. The resulting RNA pellet was washed with 75% ethanol, briefly air-dried, and resuspended in RNase-free water. RNA concentration and purity were assessed using a NanoDrop™ 2000 spectrophotometer (Thermo Fisher Scientific), with RNA purity assessed based on the A260/A280 ratio.

Following extraction, total RNA was treated with DNase I to remove residual genomic DNA contamination prior to cDNA synthesis. For each RNA sample, 5.6 μL of 10 × DNase I buffer and 1 μL of DNase I (2 U) were added to the RNA. The reaction was gently mixed by flicking and incubated at 37 °C for 20 min. DNase Inactivation Reagent (6.17 μL) was subsequently added, followed by gentle mixing and incubation at room temperature for 2 min, with intermittent mixing to ensure adequate dispersion of the reagent. Samples were then centrifuged at 10,000 × *g* for 1 min at 4 °C, and the resulting RNA-containing supernatant was transferred to a clean microcentrifuge tube. A total of 2 μg of DNase-treated RNA was subsequently used as the input for cDNA synthesis and subsequent gene expression analysis.

cDNA was synthesized from 2 μg of total RNA using the High-Capacity cDNA Reverse Transcription Kit (Thermo Fisher Scientific, South Africa, Cat. No. 4368814), according to the manufacturer's instructions. Random primers were used for reverse transcription. Briefly, the reverse transcription reaction mixture was prepared according to the manufacturer's protocol and combined with the RNA template to a final volume of 20 μL. Reactions were gently mixed, briefly centrifuged, and subjected to thermal cycling at 25 °C for 10 min, 37 °C for 120 min, and 85 °C for 5 min, followed by a hold at 4 °C. The resulting cDNA was stored at −20 °C until further use.

RT-qPCR was performed using iTaq™ Universal SYBR^®^ Green Supermix (Bio-Rad) on a Roche LightCycler^®^ 96 system. Gene expression analysis was performed for *DDC, GCH1* and *GAPDH*, which was used as the reference housekeeping gene. Primer sequences for *DDC, GCH1*, and *GAPDH* were obtained from previously published literature and are provided in Table 1 (Carmona-Carmona et al., 2025; Tatham et al., 2009). Primer specificity was verified using NCBI Primer-BLAST against human RefSeq mRNA sequences. Amplification consisted of an initial denaturation at 95 °C for 2 min, followed by 40 cycles of 95 °C for 15 s and 60 °C for 30 s, with fluorescence acquisition at the end of each cycle. A final extension step was performed at 72 °C for 10 min. Relative gene expression was calculated using the comparative ΔΔCq method.

**Table 1:** List of genes, their respective primers and accession numbers.

Gene name	Forward primer	Reverse primer	Accession numbers
*DDC*	5′ACCACAACATGCTGCTCCTTTG3′	5′CATTCAGAAGGTGCCGGAACTC3′	NM_000790
*GCH1*	5′CGCCTACTCGTCCATCCTGA3′	5′CCTTCACAATCACCATCTCA3′	NM_000161
*GAPDH*	5′ATCAGCAATGCCTCCTGCAC3′	5′TGGTCATGAGTCCTTCCACG3′	NM_002046

### 2.8 Delta-delta Ct method of analysis

Relative gene expression was determined using the ΔΔCt method as described in the MIQE guidelines (Bustin et al., 2009). The calculations were performed as follows:

- ΔCt = Ct (gene of interest) – Ct (GAPDH).
- Mean ΔCt for control group calculated.
- ΔΔCt = ΔCt (sample) – mean ΔCt (control).

### 2.9 Statistical analysis

Data are expressed as mean ± standard deviation (SD) using the ΔΔCt values. Three independent biological replicates were performed for each experimental group, with each biological replicate analyzed in technical triplicate. The mean value of the technical replicates was used for statistical analysis. The number of independent biological replicates is indicated in the corresponding figure legends. Statistical analyses were conducted using GraphPad Prism version 10 (GraphPad Software, San Diego, CA, USA). Data normality was assessed using the Shapiro–Wilk test prior to analysis. Differences between groups were evaluated using one-way analysis of variance (ANOVA) followed by Tukey–Kramer *post hoc* testing. A *p*-value < 0.05 was considered statistically significant. Owing to the absence of a time-matched 48-h 6-OHDA-only (PD control) group, direct statistical comparisons between the 48-h CRISPRa-treated groups and a 48-h PD control could not be performed.

## 3 Results

### 3.1 Effects of CRISPRa treatment on cell viability

To determine whether CRISPRa-mediated activation of *DDC* or *GCH1* affected cellular viability, an MTT assay was performed in HEK293 and SH-SY5Y cells following CRISPRa treatment. No significant differences in cell viability were observed between untreated HEK293 cells and CRISPRa-treated groups at either 24 or 48 h (Figure 1A), indicating that CRISPRa treatment did not significantly affect cell viability under the experimental conditions tested. One-way ANOVA revealed no significant differences among groups, [F(4,10) = 0.25, *p* = 0.90]. For SH-SY5Y cells (Figure 1B), ANOVA revealed a significant treatment effect, [F(5,12) = 9.63, *p* = 0.0007]. In SH-SY5Y cells, exposure to 25 μM 6-OHDA significantly reduced cell viability compared with the untreated control group (^*^p = 0.0129; Figure 1B). Cells treated with CRISPRa targeting *GCH1* also exhibited significantly lower viability than untreated controls at both 24 h (^**^p = 0.0017) and 48 h (^***^p = 0.0008). Similarly, cells treated with CRISPRa targeting *DDC* showed significantly reduced viability relative to untreated controls at 24 h (^**^p = 0.0026) and 48 h (^**^p = 0.0017). However, no significant differences were observed between the PD control group and either the *GCH1*- or *DDC*-targeted CRISPRa groups. Collectively, these findings indicate that CRISPRa treatment did not significantly alter cell viability in HEK293 cells and did not exacerbate 6-OHDA-induced cytotoxicity in SH-SY5Y cells.

**Figure 1:**
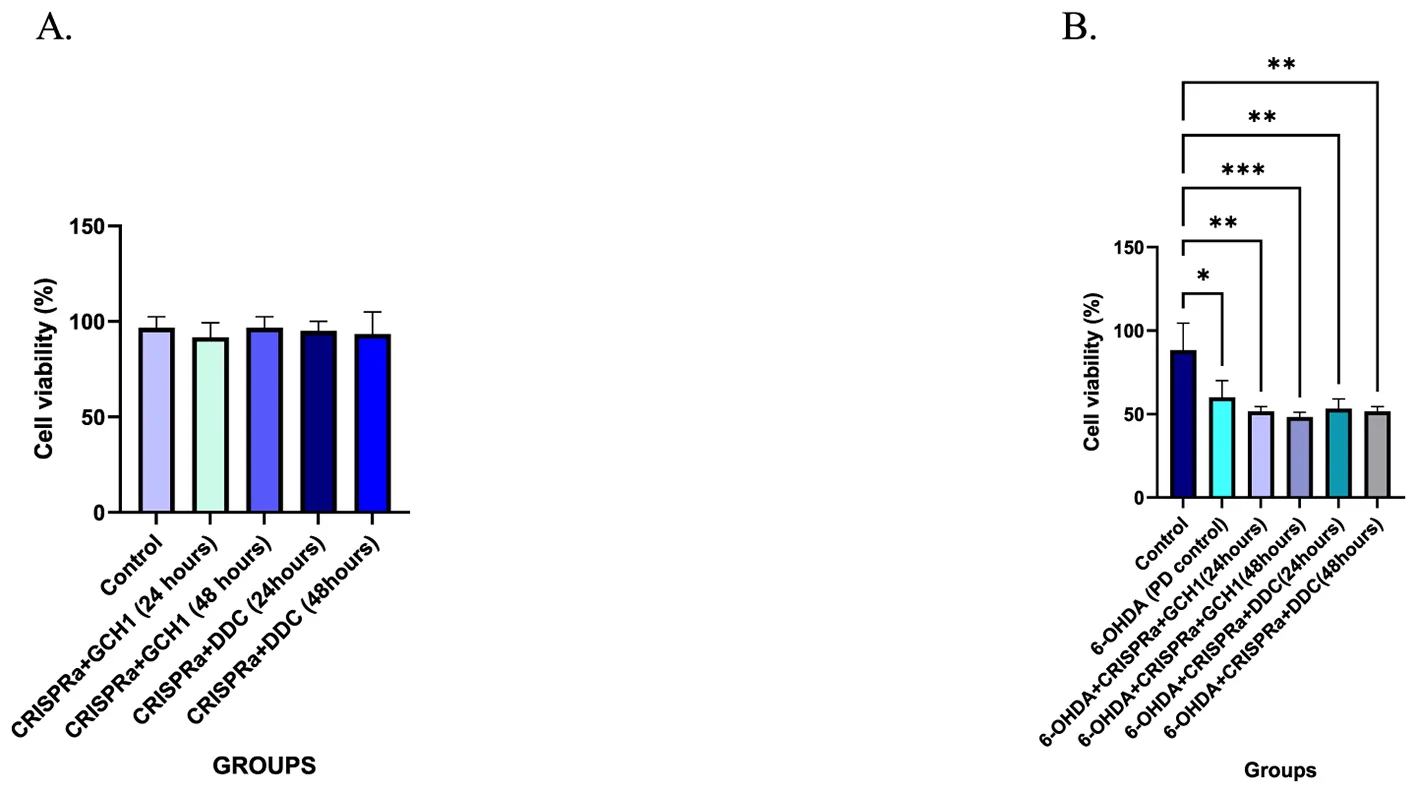
**(A)** HEK293 cells were transfected with CRISPRa complexes targeting *DDC* or *GCH1* and analysed at 24 and 48 h post-transfection. **(B)** SH-SY5Y cells were exposed to 25 μM 6-OHDA for 1 h and subsequently transfected with CRISPRa complexes targeting *DDC* or *GCH1*, with viability assessed at 24 and 48 h post-transfection. The untreated control consisted of cells that received neither 6-OHDA nor CRISPRa treatment, whereas the PD control consisted of cells treated with 6-OHDA only. Cell viability was expressed as a percentage relative to the untreated control.

Cell viability was assessed using the MTT assay following CRISPRa treatment. (A) HEK293 cells were transfected with CRISPRa complexes targeting *DDC* or *GCH1* and analyzed at 24 and 48 h post-transfection. (B) SH-SY5Y cells were exposed to 25 μM 6-OHDA and subsequently transfected with CRISPRa complexes targeting *DDC* or *GCH1*, with viability assessed at 24 and 48 h post-transfection. The untreated control consisted of cells that received neither 6-OHDA nor CRISPRa treatment, whereas the PD control consisted of cells treated with 6-OHDA only. Cell viability was expressed as a percentage relative to the untreated control. A time-matched 48-h PD control was not included; therefore, the 48-h CRISPRa-treated groups cannot be directly compared with a 48 h 6-OHDA-only control. Data are presented as mean ± SD from three independent biological replicates (*n* = 3 per group), each analyzed in technical triplicate. Statistical significance was determined using one-way ANOVA followed by Tukey–Kramer *post hoc* testing. ^*^p < 0.05, ^**^p < 0.01, ^***^p < 0.001, and ^****^p < 0.0001.

### 3.2 Gene expression

#### 3.2.1 Effects of CRISPRa on GCH1 and DDC expression in HEK293 cells

To evaluate whether CRISPRa could activate endogenous *GCH1* and *DDC* expression in HEK293 cells, gene expression was quantified by RT-qPCR following transfection (Figure 2). For *GCH1* expression (A), no significant treatment effect was observed [one-way ANOVA, F(2,6) = 0.58, *p* = 0.59]. For *DDC* expression (B), no significant treatment effect was observed [one-way ANOVA, F(2, 6) =2.73, *p* = 0.14]. No significant differences in *GCH1* expression were observed between CRISPRa-treated groups and untreated controls at either 24 or 48 h post-transfection (Figure 2A). Similarly, *DDC* expression did not differ significantly between CRISPRa-treated groups and the untreated control, and no significant differences were observed between the 24- and 48-h treatment groups (Figure 2B). Overall, CRISPRa targeting of *GCH1* and *DDC* did not result in detectable transcriptional activation in HEK293 cells under the experimental conditions tested.

**Figure 2:**
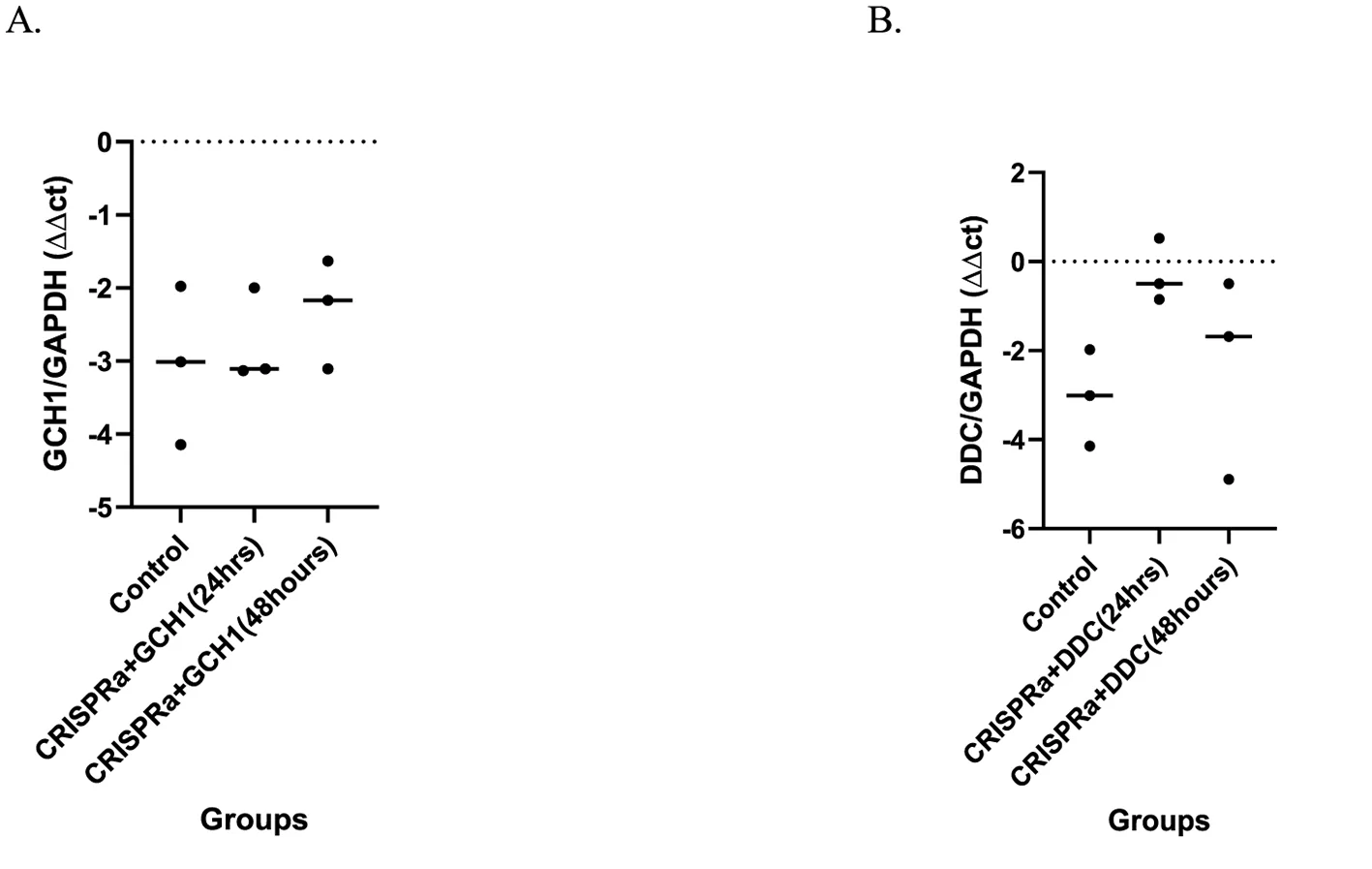
**(A)** Relative *GCH1* expression were measured by RT-qPCR following CRISPRa transfection of HEK293 cells. Cells were either left untreated (Control) or transfected with CRISPRa complexes targeting *GCH1* and analysed at 24 and 48 h post-transfection. Gene expression was normalised to *GAPDH* and calculated using the comparative ΔΔCt method relative to the untreated control group. **(B)** relative *DDC* expression were measured by RT-qPCR following CRISPRa transfection of HEK293 cells. Cells were either left untreated (Control) or transfected with CRISPRa complexes targeting *DDC* and analysed at 24 and 48 h post-transfection. Gene expression was normalised to *GAPDH* and calculated using the comparative ΔΔCt method relative to the untreated control group.

(A) Relative *GCH1* expression and (B) relative *DDC* expression were measured by RT-qPCR following CRISPRa transfection of HEK293 cells. Cells were either left untreated (Control) or transfected with CRISPRa complexes targeting *GCH1* or *DDC* and analyzed at 24 and 48 h post-transfection. Gene expression was normalized to *GAPDH* and calculated using the comparative ΔΔCt method relative to the untreated control group. Data are presented as mean ± SD from three independent biological replicates (*n* = 3 per group), each analyzed in technical triplicate. Individual data points represent independent biological replicates, the central bar represents the mean, and error bars indicate the SD. Statistical significance was assessed using one-way ANOVA followed by Tukey-Kramer multiple-comparison testing. *p* < 0.05 was considered statistically significant. Significance is indicated as ^*^p < 0.05, ^**^p < 0.01, ^***^p < 0.001, and ^****^p < 0.0001.

#### 3.2.2 Effects of CRISPRa targeting of GCH1 and DDC on gene expression in SH-SY5Y cells

To assess the transcriptional effects of CRISPRa-mediated targeting of *GCH1* and *DDC* in a Parkinsonian cell model, relative gene expression was quantified by RT-qPCR in 6-OHDA-treated SH-SY5Y cells (Figure 3). No significant differences in *GCH1* expression were observed between CRISPRa-treated groups and either the untreated control or PD control groups [one-way ANOVA, **F**(3, 5) = 4.22, *p* = 0.078] (Figure 3A). Similarly, no significant differences were detected between the 24 and 48 h *GCH1*-targeted treatment groups. These findings indicate that CRISPRa targeting of *GCH1* did not result in detectable transcriptional activation under the experimental conditions tested. In contrast, *DDC* expression differed significantly among treatment groups [one-way ANOVA, **F**(3,6) = 12.63, *p* = 0.0053] (Figure 3B). *Post hoc* Tukey-Kramer analysis showed significantly increased *DDC* expression in CRISPRa-*DDC*-treated cells relative to the untreated control at 24 h (^*^p = 0.0496) and 48 h (^**^p = 0.0064). The PD control group also exhibited significantly different *DDC* expression relative to the untreated control (^*^p = 0.0249). However, no significant differences in *DDC* expression were observed between the CRISPRa-*DDC* groups and the PD control at either time point. Collectively, these findings indicate a modest transcriptional response following CRISPRa targeting of *DDC*, whereas no detectable transcriptional activation of *GCH1* was observed in SH-SY5Y cells under the conditions tested.

**Figure 3:**
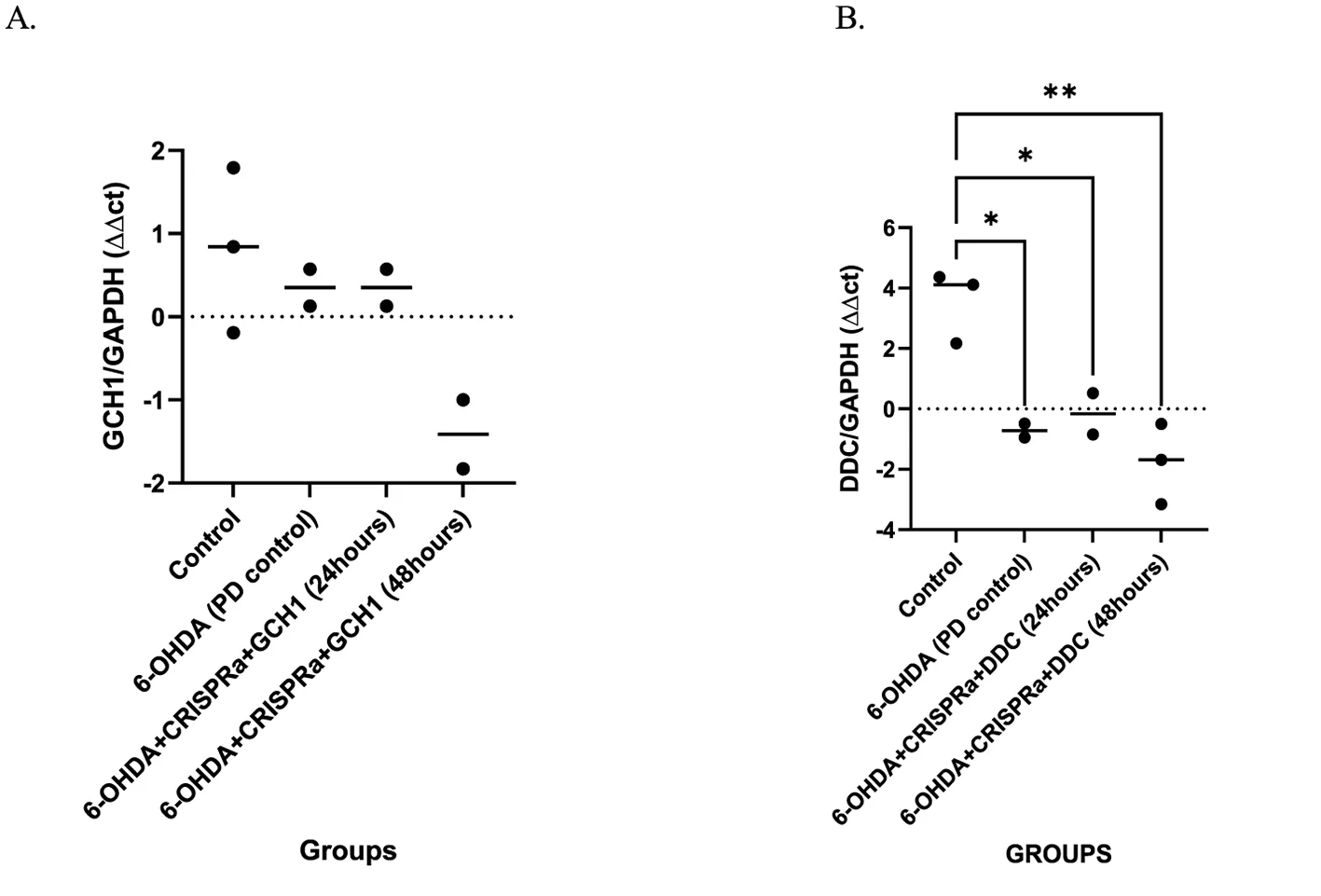
**(A)** Relative *GCH1* expression was quantified by RT-qPCR in SH-SY5Y cells following exposure to 25 μM 6-OHDA and subsequent CRISPRa treatment. Gene expression was normalised to *GAPDH* and calculated using the comparative ΔΔCt method relative to the untreated control group. **(B)** Relative *DDC* expression was quantified by RT-qPCR in SH-SY5Y cells following exposure to 25 μM 6-OHDA and subsequent CRISPRa treatment. Gene expression was normalised to *GAPDH* and calculated using the comparative ΔΔCt method relative to the untreated control group.

Relative *GCH1* (A) and *DDC* (B) expression was quantified by RT-qPCR in SH-SY5Y cells following exposure to 25 μM 6-OHDA and subsequent CRISPRa treatment. Gene expression was normalized to *GAPDH* and calculated using the comparative ΔΔCt method relative to the untreated control group. Data are presented as mean ± SD. Biological replicate numbers were as follows: Control (*n* = 3), PD control (*n* = 3), CRISPRa-*DDC* 24 h (*n* = 2), CRISPRa-*DDC* 48 h (*n* = 3), CRISPRa-*GCH1* 24 h (*n* = 2), and CRISPRa-*GCH1* 48 h (*n* = 3). Each biological replicate was analyzed in technical triplicate, and the mean value was used for statistical analysis. Individual data points represent independent biological replicates, the central bar represents the mean, and error bars indicate the SD. Statistical significance was assessed using one-way ANOVA followed by Tukey-Kramer *post hoc* testing. Significance is indicated as ^*^p < 0.05, ^**^p < 0.01, ^***^p < 0.001, and ^****^p < 0.0001.

## 4 Discussion

This study investigated the effects of CRISPRa-mediated targeting of *GCH1* and *DDC* expression in HEK293 cells and 6-OHDA-treated SH-SY5Y cells. The findings provide preliminary evidence of cellular and transcriptional responses to this approach.

In HEK293 cells, no significant differences in cell viability were observed between control and CRISPRa-treated groups (Figure 1A), indicating that the treatment did not significantly compromise cell viability under the conditions tested. HEK293 cells were primarily included as a transfection-compatible model to assess cellular responses to CRISPRa treatment and were not intended to represent a dopaminergic cellular environment. In SH-SY5Y cells, viability was reduced in the PD control and CRISPRa-treated groups relative to untreated controls (Figure 1B), consistent with the cytotoxic effects of 6-OHDA. Importantly, CRISPRa treatment did not further reduce viability relative to the PD control, indicating that targeting *DDC* or *GCH1* did not significantly compromise cell viability beyond that associated with 6-OHDA exposure. However, these findings cannot be taken as evidence of successful CRISPRa delivery or activation, as transfection efficiency and CRISPRa activity were not independently quantified.

In SH-SY5Y cells, CRISPRa targeting of *GCH1* did not significantly increase expression (Figure 3A). Although the overall analysis was not statistically significant [F(3,5) = 4.22, *p* = 0.078], the observed pattern of expression suggests possible variability between treatment groups that warrants further investigation under conditions of greater statistical power. The relatively small number of biological replicates in some treatment groups, particularly the CRISPRa-*GCH1* 24 h group (*n* = 2), may have reduced the ability to detect subtle changes in expression. In addition, limited delivery or activation of the CRISPRa components, differences in chromatin accessibility, gene-specific regulatory mechanisms, or cellular context may have influenced responsiveness. However, these factors were not directly assessed in the present study. Consequently, while the findings do not provide evidence of significant *GCH1* activation, they suggest that further investigation with increased replication and optimisation of CRISPRa delivery may be warranted to determine whether biologically meaningful modulation of *GCH1* expression can be achieved.

For *DDC*, CRISPRa-targeted cells exhibited increased expression relative to untreated controls, but expression did not differ significantly from the PD control (Figure 3B), indicating only limited evidence of target activation under the conditions assessed. The increase observed at 48 h may represent a transcriptional response to CRISPRa treatment; however, because expression levels were not significantly different from those of the PD control group, the contribution of CRISPRa-mediated activation cannot be clearly distinguished from transcriptional changes associated with 6-OHDA exposure. The significant difference between untreated and PD control cells further suggests that 6-OHDA altered baseline *DDC* expression, although the underlying mechanism was not investigated.

The relatively modest transcriptional responses observed in the present study contrast with reports demonstrating robust activation of endogenous genes using CRISPRa-based transcriptional activators. Early CRISPRa studies demonstrated that recruitment of transcriptional activation domains to catalytically inactive Cas9 (dCas9) can produce substantial increases in endogenous gene expression. For example, Gilbert et al. (2014) demonstrated targeted transcriptional activation of endogenous genes using dCas9-based activator systems, while Konermann et al. (2015) reported synergistic gene activation using the Synergistic Activation Mediator (SAM) CRISPRa system. Similarly, Chavez et al. (2015) showed that the tripartite VPR activator substantially enhanced transcriptional activation across multiple endogenous targets (Chavez et al., 2015; Konermann et al., 2015; Gilbert et al., 2014). These studies demonstrated that the magnitude of CRISPRa-mediated activation is highly dependent on factors such as sgRNA design, chromatin accessibility, target locus architecture, and delivery efficiency. In comparison, *DDC* expression in the present study was elevated relative to untreated controls, whereas *GCH1* expression showed only a non-significant trend toward increased expression. These comparatively limited responses may reflect suboptimal target engagement, reduced accessibility of the targeted genomic regions, or insufficient intracellular delivery of the CRISPRa ribonucleoprotein complexes. Furthermore, many studies reporting strong CRISPRa-mediated activation incorporated direct validation of transfection efficiency and target engagement, thereby allowing transcriptional changes to be confidently attributed to CRISPRa activity. In contrast, the absence of such validation in the current study makes it difficult to distinguish between limited biological responsiveness and suboptimal CRISPRa delivery. Consequently, while the findings provide preliminary evidence of transcriptional modulation, further optimisation and validation will be required to determine whether more substantial activation of *DDC* and *GCH1* can be achieved in neuronal cell models.

The magnitude of the transcriptional responses may also have been influenced by the concentration and duration of 6-OHDA exposure. A concentration of 25 μM for 1 h was selected following preliminary optimisation and produced measurable loss of SH-SY5Y viability while retaining sufficient viable cells for subsequent analysis. The *DDC* response observed at 48 h may therefore represent a transcriptional response to oxidative stress. However, stress-response pathways were not measured, and additional time-course studies incorporating direct measures of oxidative stress and cellular signaling would be required to determine their contribution.

Differences in CRISPRa responsiveness between HEK293 and SH-SY5Y cells may reflect their distinct cellular contexts. HEK293 cells are non-neuronal and generally amenable to transfection, whereas SH-SY5Y cells possess neuronal characteristics and are more relevant to dopamine-associated pathways. Differences in basal gene expression, transcriptional regulation, and chromatin accessibility could therefore influence CRISPRa responsiveness. Similarly, the temporal changes in *GCH1* and *DDC* expression observed in HEK293 cells cannot be confidently attributed to target-specific CRISPRa activity because target activation was not independently validated. Cell-specific regulation, temporal variation, or experimental variability may have contributed to these observations (Figures 2A and B).

The functional significance of the observed mRNA changes remains uncertain. *DDC* encodes AADC, which catalyzes the conversion of L-dopa to dopamine, while *GCH1* contributes to BH_4_ biosynthesis and thereby supports TH activity within the dopamine biosynthetic pathway. Because dopamine biosynthesis depends on the coordinated activity of multiple pathway components, modulation of a single component may be insufficient to produce a measurable increase in dopamine production. Moreover, increased mRNA abundance does not necessarily result in increased protein expression or enzyme activity. Because protein, enzymatic, dopamine, and related metabolite measurements were not performed, the present findings cannot establish a functional alteration in dopamine biosynthesis or provide evidence of dopaminergic rescue or therapeutic efficacy.

Several limitations should therefore be considered. CRISPRa delivery and activation were not quantitatively validated, and the study lacked non-targeting sgRNA and transfection-only controls, limiting the ability to distinguish target-specific effects from effects associated with transfection or CRISPRa components. CRISPRa responsiveness may also have been influenced by sgRNA design and target-site accessibility, neither of which was experimentally validated. The use of three biological replicates per group may have limited statistical power to detect modest transcriptional effects; consequently, small but biologically meaningful changes may not have been detected. Due to technical failures during RT-qPCR analysis, some groups contained fewer biological replicates than originally planned. This reduction in sample size may have limited statistical power and increased the likelihood of failing to detect modest transcriptional effects. Finally, although SH-SY5Y cells provide a useful neuronal-like model, they do not fully reproduce the molecular and cellular complexity of human dopaminergic neurons.

Future studies should incorporate quantitative assessment of CRISPRa delivery and target activation together with appropriate non-targeting and transfection controls. Protein-level validation and measurement of AADC and GCH-1 enzymatic activity would establish whether transcriptional changes translate into altered protein function, while direct quantification of dopamine and related metabolites would determine whether these changes influence dopamine biosynthesis. Optimisation of sgRNA design and CRISPRa activation, together with coordinated targeting of multiple components of the dopamine biosynthetic pathway, may further improve the approach. Validation in more physiologically relevant models, including iPSC-derived dopaminergic neurons, would also determine whether the responses observed in SH-SY5Y cells are reproducible in a representative neuronal context.

In summary, CRISPRa targeting of *DDC* and *GCH1* did not significantly compromise cell viability in HEK293 or 6-OHDA-treated SH-SY5Y cells under the conditions tested. Transcriptional effects were limited, with no significant *GCH1* upregulation and increased *DDC* expression relative to untreated controls but not the PD control. Because CRISPRa delivery and activation were not independently quantified and downstream functional measurements were not performed, restoration of dopaminergic biosynthesis cannot be established. The findings therefore provide preliminary evidence of cellular and transcriptional responses to CRISPRa targeting and highlight the key experimental validation steps required before conclusions regarding therapeutic potential can be drawn.

## Data Availability

The datasets generated and/or analysed during the current study are available in the Figshare repository at: https://doi.org/10.6084/m9.figshare.34012407. The deposited dataset includes the raw MTT assay measurements and the RT-qPCR data underlying the findings reported in this study.
